# Medical Department of Cincinnati College

**Published:** 1837

**Authors:** 


### III.—ORIGINAL.

---

### THE WESTERN JOURNAL.

---

*E Sylvis Nuncius.*

---

CINCINNATI, APRIL 1st, 1837.

---

### ART. I.—THE MEDICAL DEPARTMENT OF THE CINCINNATI COLLEGE.

The second session of this department closed on the last Saturday of February. The following catalogue of its pupils, is extracted from the annual publication of the Board of Trustees.

CATALOGUE.

| NAMES. | RESIDENCE. | PRIVATE PRECEPTORS. |
|---|---|---|
| | OHIO. | |
| John Bamford, | *Muskingum county,* | Practitioner. |
| Moses Parry Baird, | *Ripley,* | Dr. Beasley. |
| William J. Barbee, M. D. | *Cincinnati,* | |
| Ensign S. Billings, | *Cincinnati,* | Practitioner. |
| William M. Charters, | *Lebanon,* | Practitioner. |
| William Pitt Cushman, | *Lorain county,* | |
| Samuel Davis, | *Lorain county,* | |
| Eli Dayton, | *Cincinnati,* | Prof. McDowell. |
| Josiah Martin Dillon, | *Monroe county,* | Practitioner. |
| Ezer Dillon, | *Muskingum county,* | Practitioner. |

| | | |
|---|---|---|
| John Edwin Ely, | *Cincinnati,* | Prof. Gross. |
| Benjamin Evans, | *Waynesville,* | Dr. Fisher. |
| John Evans, | *Waynesville,* | Dr. Fisher. |
| Isaac Fisher, | *Waynesville,* | Practitioner. |
| Albert M. Fletcher, | *Cincinnati,* | |
| George Osgood Hildreth M. D. | *Marietta,* | |
| Smith Holloway, | *Flushing,* | Dr. Alexander. |
| Rawlah Holson, | *Hamilton county,* | Prof. Gross. |
| Thomas P. Hotchkiss, M. D. | *Cincinnati,* | |
| Edward Kimball, | *Cincinnati,* | Prof. McDowell. |
| James Ward Lewis, | *Springfield,* | Dr. Towler. |
| George Light, | *Cincinnati,* | Prof. McDowell. |
| Samuel M. McIlhenny, | *Brown county,* | Dr. Beasley. |
| Robert R. McIlwayne, | *Cincinnati,* | |
| John Miller, | *Clermont county,* | Practitioner. |
| Jacob C. Nicholson, | *Miami county,* | Prof. McDowell. |
| James W. M. Quinn, | *Hillsborough,* | Dr. Davis. |
| Charles Reed, M. D. | *Cincinnati,* | |
| Silas Reed, M. D. | *Fulton,* | |
| Edward Sheil, | *Oxford,* | Dr. Bonner. |
| Aaron G. Stout, | *Hamilton county,* | Dr. Gano. |
| Isaac Thorne, | *Jefferson county,* | Dr. Parker, |
| Cary A. Trimble, M. D. | *Hillsborough,* | |
| Aquila Toland, | *Madison county,* | Practitioner. |
| David Wilson, | *Morgan county,* | Dr. Dodge. |
| Samuel R. Wilson, | *Cincinnati,* | |
| John A. Young, | *Xenia,* | Dr. Martin. |

### KENTUCKY.

| | | |
|---|---|---|
| John S. Alexander, | *Woodford county,* | Dr. Carter. |
| Joseph J. H. Grady, | *Logan county,* | Prof. Harrison. |
| John H. Grant, | *Campbell county,* | Prof. Gross. |
| John Hann, | *Lancaster,* | Dr. Letcher. |
| William Peyton, | *Hartford,* | Dr. Peyton. |
| David W. Rannells, | *Washington,* | Dr. Clarkson. |
| Harrison Symmes, | *Frankfort,* | Prof. McDowell. |
| Benjamin B. Thornton, | *Paris,* | Dr. Lindsey. |
| George A. Williams, | *Logan county,* | Practitioner. |
| Theodore N. Wise, | *Newport,* | Dr. Bennet. |
| Isaac H. Wright, | *Mason county,* | Dr. Sharpe. |

### MISSISSIPPI.

| | | |
|---|---|---|
| Joseph H. Anderson, | *Vicksburg,* | Prof. Harrison. |
| John Calderwood, | *Yazoo county,* | Prof. McDowell. |
| William M. Dalton, | *Woodville,* | Prof. Gross. |
| Benjamin Dennis, | *Madison county,* | Prof. Harrison. |
| Thomas P. Harrison, | *Wilkinson county,* | Dr. Dodge. |
| James O. Hutchins, | *Clabourne county,* | |
| Charles G. Trask, | *Woodville,* | Dr. Wood. |
| Benjamin J. Woodward, | *Hinds county,* | Prof. Gross. |

### VIRGINIA.

| | | |
|---|---|---|
| T. J. Bicknell, | *Wheeling,* | Dr. Tanner. |
| Edward O. Goodwyn, | *Petersburg,* | Prof. McDowell. |
| J. P. Morgan, M. D., | | |
| John Parkes, | *Charleston,* | Prof. Rives. |
| Alexander A. Patteson, | *Prince Edward,* | Dr. Christian. |
| Lucien M. Watkins, | *Goochland county,* | Prof. Rives. |
| William A. Wilkinson, | *Rockbridge county,* | Dr. Leyburn. |

### ALABAMA.

| | | |
|---|---|---|
| J. P. Epperson, | *Huntsville,* | Drs. Fearn and Erskine. |
| William J. P. Haughton, | *Huntsville,* | Dr. Wharton. |
| John W. King, | *Huntsville,* | Drs. Fearn and Erskine. |
| Achilles Whitlocke, | *Limestone county,* | Practitioner. |
| James D. Wiley, | *Madison county,* | Dr. Wood. |

### INDIANA.

| | | |
|---|---|---|
| Robert Cogley, | *Union county,* | Practitioner. |
| Thomas W. Colescott, | *Brookville,* | Dr. Haymond. |
| William R. Winton, | *Crawfordsville,* | Practitioner. |

### NEW YORK.

| | | |
|---|---|---|
| Martin C. Collins, | *Rochester.* | Prof. Gross. |
| Samuel D. Dodge, | *City of New York.* | Dr. Dodge. |
| Alexander Duncan, | *City of New York.* | |

### MISSOURI.

| | | |
|---|---|---|
| Moses C. Cooke, | *St. Louis,* | Dr. Vanzandt. |
| Bartley W. Gorin, | *Pike county,* | Prof. Gross. |
| Silas McDonald, | *Howard county,* | Dr. Tomlinson. |

### ILLINOIS.

| | | |
|---|---|---|
| Francis W. Todd, | *Springfield,* | Dr. Todd. |
| William C. Stephenson, | *Sangamo county,* | Dr. Henry. |

### MICHIGAN.

| | | |
|---|---|---|
| Horace B. White, | *Calhoun county,* | Dr. Greves. |

### NEW ENGLAND.

| | | |
|---|---|---|
| Benjamin F. Clark, | *New Hampshire,* | |
| Josiah B. Clark, | *New Hampshire.* | |
| Charles P. Gage, | *New Hampshire.* | Prof. Parker. |
| Jesse Caswell. | *Vermont,* | |
| Daniel H. Dickenson, | *Massachusetts,* | Drs. Childs and Parker. |
| | | Total, 85 |

### GRADUATES.

The commencement for conferring the degree of Doctor of Medicine, was held in the Hall of the College, on the 3d of March, in the presence of a very numerous audience of ladies and gentlemen; when the following *alumni* received diplomas:

### OHIO.

John Bamford, Thesis on Spinal Irritation.
William B. Charters, On Sanguineous Congestion.
Ezer Dillon, On Inflammation.
Joseph M. Dillon, On Pathology.
Benjamin Evans, On Mental Alienation.
Isaac Fisher, On the Uses and Abscesses of Cathartics.
Edward Shiel, On the Healthy and Morbid Aantomy of the Lymphatic Gauglia.
Aqula Toland, On Wounds of the Intestines.

### KENTUCKY.

Joseph J. H. Grady, On Inflammation.
John Hann, On Pleuritis.
David W. Rannels, On Alterations of the Blood.
Harrison Symmes, On Intermittent Fever.
George A. Williams, On Intermittent Fever.
Theodore N. Wise, On Intermittent Fever.

### MISSISSIPPI.

Joseph H. Anderson, On the Use of Cold Water in Fevers.
William M. Dalton, On the Blood.
Benjamin Dennis, On Hysteria.
Thomas P. Harrison, On Digestion.
Charles G. Trask, On some of the promising causes of Disease.
Benjamin J. Woodward, On Tetanus.

### INDIANA.

Robert Gogley, On Bloodletting.
William R. Winton, On Dysmenorrhœa.

### MISSOURI.

Bartley W. Gorin, On Inguin Hernia.
Silas McDonald, On the Modus Operandi of Medicines.

### ALABAMA.

Achilles Whitlock, On the Cold Affusion in Congestive Fever.

### NEW YORK.

Martin C. Collins, On the Healthy and Morbid Anatomy of the Mucous Membranes.

### NEW HAMPSHIRE.

Charles P. Gage, On Necrosis.

Of these graduates, two had attended a previous course of lectures in the Medical College of Ohio; two in the University of Pennsylvania; one in the Berkshire Medical Institute; and five in Transylvania University; the remaining seventeen in the Cincinnati College only.

The honorary degree of Doctor of Medicine, was at the same time conferred on Dudley W. Rhodes and Hezekiah Bissell, of the State of Ohio.

### FACULTY.

JOSEPH N. McDOWELL, M. D.
*Professor of Special and Surgical Anatomy.*

SAMUEL D. GROSS, M. D.
*Professor of General and Pathological Anatomy and Physiology.*

WILLARD PARKER, M. D.
*Professor of Surgery.*

LANDON C. RIVES, M. D.
*Professor of Obstetrics and the Diseases peculiar to Women and Children.*

JAMES B. ROGERS, M. D.
*Professor of Chemistry and Medical Jurisprudence, and Dean of the Faculty.*

JOHN P. HARRISON, M. D.
*Professor of Materia Medica and Pharmacy.*

DANIEL DRAKE, M. D.
*Professor of the Theory and Practice of Medicine.*

CARY A. TRIMBLE, M. D.
*Anatomical Demonstrator.*

EDWARD KIMBALL,
*Clinical Clerk and House Surgeon, of the Cincinnati Hospital.*

### TO MEDICAL STUDENTS.

### Arrangements for Summer Instruction.

#### ANATOMY.

Immediately after the close of the session, on the first of March, the rooms for practical anatomy were opened by the *Demonstrator*, Dr. Trimble, and will be kept open for six weeks. On the middle of September, they will be re-opened, and every facility for the prosecution of practical Anatomy afforded, till the last Monday of October. At the beginning of that month, Professor McDowell will commence a preliminary course of Osteology.

#### Clinical Instruction.

The Cincinnati Hospital is the Clinical school of the Cincinnati College. From the first of March to the first of November, every day, except Sundays, will be a prescribing day, from 9 to 10 in the morning, and twice a week, the physician and surgeon in attendance will, each, deliver a clinical lecture.

### Practical Chemistry.

In the summer, Professor Rogers, will deliver a familiar course of instruction on Mineral Toxicology, and the analysis of Mineral Waters; in which the students will be permitted to assist in the manipulations, so as to acquire a practical facility in the application of antidotes and re-agents.

### Mental Physiology.

At some convenient period of the vacation, President McGuffey will deliver a short series of lectures on Mental Physiology, in its application to the medical profession.

### Reading.

The Library of the College will be open for the delivery of books every Wednesday and Saturday afternoon, and each student will be allowed to take out two volumes at once.

### Expenses.

The aggregate cost of these advantages will be thirty dollars, but the student may avail himself of all, or any one of them, at his pleasure.

### Arrangements for the Ensuing Winter.

The seven professors, now in the institution, are regarded as permanent incumbents; Professor Parker having accepted the chair of Surgery, and resolved on a removal to this city. He is gone to Euope and will return with books, chemical apparatus, surgical instruments, and anatomical preparations, before the commencement of the next session, when the means of instruction will be greatly augmented.

The session will open, as before, on the last Monday in October, and continue to the end of February, during which six lectures will be delivered daily.

The professors of Surgery, and the Theory and Practice of Medicine, will attend at the Hospital three times a week, from two to three o'clock in the afternoon; when all the candidates for graduation will be expected to be present; as it is required of them to take the Hospital ticket and attend the practice.

The lectures on Pathological Anatomy, will be chiefly demonstrative, as professor Gross, has already collected more than 400 morbid specimens, and is, from various quarters, augmenting the number almost daily.

President McGuffey, will deliver a course of Lectures on the Physiology of the Mind, at such hours as will not interfere with the lectures by the medical professors, and the students of medicine will be entitled to attend them.

The Cincinnati Medical Society holds its meetings weekly, in the College Edifice; and the students of the institution are admitted to the discussions.

The Medical Library of the College, when the purchases making by prof. Parker shall be added to those now on hand, will contain about

2000 volumes, of which the students may avail themselves or not, as they please.

The dissecting room will be kept open throughout the session, under the superintendence of Dr. Trimble; and every candidate is required to take the ticket once, prior to graduation.

The total cost of all the tickets (including the Library, which is left optional with the student) is one hundred and twenty-five dollars.

Comfortable summer boarding and lodging may be had for two dollars and fifty cents a week: winter accommodations, including light and fuel, for three dollars.

Students on arriving in the city, may obtain every desirable information, by applying to the resident physician of the Cincinnati Hospital opposite the College Edifice, corner of Fourth and Walnut streets.

### PROFESSOR PARKER.

We feel it a duty to the profession in the West, not less than to the Cincinnati College, to say, that Prof. Parker, in his course of surgical lectures last winter, gave to the class a degree of satisfaction that we have never known surpassed by any professor, in any branch of the profession. His eloquence is admirably adapted to the lecture room, and he possesses the rare ability, of speaking and operating, simultaneously, so as to address both the ear and eye of the pupil. At the same time, his knowledge both theoretical and practical, is profound and accurate. Prof. Parker stands very high as a teacher, in the eastern states, but when appointed into the Cincinnati College was little known, in the west and south,—a fact that justifies this notice. He came to Cincinnati on probation, and to study the prospects of her medical school, before he finally dissolved his connexion with the eastern institutions, to which he had for several years been attached. Having satisfied himself on all points connected with the Cincinnati College, and more than fulfilled the expectations of its Board of Trustees and Medical Faculty, he proceeded to lecture twice a day, that he might depart on the first of February for Europe. On the first of March he sailed in the Charlemagne from New York for France; and will return in Autumn, with several thousand dollars worth of books, apparatus and preparations; for the purchase of which he has been furnished with the requisite means, by the Medical

Faculty. In a future number of the Journal we hope to lay before our readers, some of his European observations.

CLINICAL INSTRUCTION.

The Hospital for Clinical instruction is on the opposite side of the street from the College Edifice. The prescribing hour is from two to three in the afternoon, three times a week when there is no lecture. Although established at the very opening of the last session, we are enabled to say, that it presented, to the pupils a great variety of interesting cases, including several surgical operations. It is not a *poor-house*, and, therefore, every inmate furnishes a clinical study. Many of the patients were affected with maladies of the eye; as the *Cincinnati Eye Infirmary*, has been united with the Hospital; and all the operations that are any where performed on the eye, will be performed in the hospital, if occasion should require.

In the next number of the Journal we shall have a series of Hospital reports; and the first article of every succeeding number, will be of the same kind.

PROSPECTS OF THE SCHOOL.

If we are not misinformed, no medical institution in the United States, before this, has had 85 professional pupils in its *second* session; and none, ever before, at its *second* commencement, had 27 graduates. Such prosperity has raised the new school; from an experiment into a reality, and destined it, we cannot doubt, to take rank, forthwith, among the most respectable institutions of the Union. It must and will be permanent. The professors alone have already expended on the object more than twelve thousand dollars; and by the ensuing autumn, their accommodations and various means of instruction, will be as ample, as the unyielding resolution with which they are prosecuting an enterprize—to which they have dedicated their lives.

